# The impact of motivation and teachers’ autonomy support on children’s executive functions

**DOI:** 10.3389/fpsyg.2015.00146

**Published:** 2015-02-13

**Authors:** Zrinka Sosic-Vasic, Oliver Keis, Maren Lau, Manfred Spitzer, Judith Streb

**Affiliations:** ^1^Department for Psychiatry and Psychotherapy, University Clinic of Ulm, UlmGermany; ^2^Transfercenter of Neuroscience and Learning, University of Ulm, UlmGermany; ^3^Department of Forensic Psychiatry and Psychotherapy, University of Ulm, UlmGermany

**Keywords:** motivation, autonomy, self-regulation, executive functions, school

## Abstract

The present study investigates the interplay of executive functions, motivation, and teacher’s autonomy support in school context. In a cross-sectional study design 208 students from different school types completed a standardized motivation questionnaire and processed two executive function tasks. All teachers who teach these students were asked about their autonomy supporting behavior by a standardized test. Multilevel analyses assessed the effects of the student’s motivation and their teachers’ autonomy support on student’s executive functions. Our results show considerable relationships between these variables: high executive function capacities came along with teacher’s autonomy support and student’s intrinsic motivation styles, whereas low executive function capacities were related to external regulation styles. The results indicate the importance of autonomy support in school instruction and disclose the need to popularize the self-regulation approach.

## INTRODUCTION

Cognitive abilities influence the way children succeed in school. For example, low levels of cognitive abilities at school entry are associated with lower later academic achievement, greater retention and special education referral, and furthermore have the potential to enhance dropout rates in school (Carnegie Council on Adolescent Development, 1995). Among different cognitive abilities, the so-called intelligent quotient (IQ) is one of the most empirically investigated factor influencing learning. However, in recent years there has been a growing body of evidence indicating that other cognitive factors such as executive functions may play a role in learning during childhood and adolescence (Swanson, 1993; Lehto, 1995; Lorsbach et al., 1996; Russell et al., 1996; Swanson et al., 1996; Bull et al., 1999; McLean and Hitch, 1999; Ozonoff and Jensen, 1999; Duckworth and Seligman, 2005; St Clair-Thompson and Gathercole, 2006). Executive functions can be understood as a set of separate cognitive components interplaying and operating together in response to cognitive and behavioral demands requiring self-regulation. Converging research suggests that executive functions may be best conceptualized as consisting of three separable, yet related dimensions: working memory (Miyake et al., 2000; Huizinga et al., 2006), inhibition (Welsh et al., 1991; Pennington and Ozonoff, 1996; Brocki and Bohlin, 2004), and cognitive flexibility (Miyake et al., 2000; Huizinga et al., 2006; van der Sluis et al., 2007). However, many researchers have used the term shifting instead of (cognitive) flexibility (e.g., Miyake et al., 2000) and have differentiated two kinds of shifting, namely attention shifting and response shifting. Together, these three cognitive processes build a fundamental basis for self-regulation and therefore may represent important predictors for academic achievement. For example, if a child has impairments in working memory, it may have trouble remembering and following teacher directions or memorizing and recalling task-related information when needed. Or, if a child has impairments in inhibition, it may have trouble focusing on given tasks without being constantly distracted by other (sometimes more appealing) impulses, or, such as child may fail to inhibit socially non-desirable behaviors in its learning environment such as kindergarten and school classroom. Yet, another example, if a child has impairments in cognitive flexibility/shifting, it may get stuck on a thought, perseverating only on that topic, even though other impulses are given that might lead to the appropriate direction.

Cumulating research data supports the idea of executive functions serving as a key predictor of academic achievement. Most studies have focused on executive functions in preschoolers, showing that those preschoolers with strong executive functions achieve higher levels of literacy, vocabulary, and mathematics compared with children with lower executive functions (Espy et al., 2004; Blair and Razza, 2007; McClelland et al., 2007). Other research suggests that executive functions skills may enhance the school readiness of children in general, as well as from disadvantaged homes (for review see Blair, 2002; Fuhs and Day, 2011). Even though comparable sparse, some research has examined the relation between aspects of executive functions and school achievement. For example, cognitive flexibility is associated with non-verbal reasoning and reading in 9- to 12-years old school children (van der Sluis et al., 2007), while working memory was found to be uniquely associated with reading comprehension in 9- to 15-years old school children (Sesma et al., 2009). Other groups again have tied working memory to reading and math skills in elementary students (Kail, 2003). Furthermore, previous findings have reported associations between working memory skills and national curriculum assessments of English, mathematics, and science in English school children (Gathercole and Pickering, 2000a,b; Gathercole et al., 2003, 2004; Jarvis and Gathercole, 2003; St Clair-Thompson and Gathercole, 2006). Over all, working memory skills are found to play a substantial role for all or some of the skills assessed by English tests: reading (e.g., Swanson et al., 2004) and writing (see Swanson and Berninger, 1994, 1995 for a review). The development of executive functions in children and adolescents is reasonably well understood (e.g., Carlson, 2005). Several studies indicate an age-related gain in executive functions such as working memory (Casey et al., 2000; Vuontela et al., 2003), inhibition (van der Molen, 2000), cognitive flexibility like task switching (Cepeda et al., 2001), adaptive problem-solving (Chelune and Baer, 1986), and several other planning and problem-solving tasks (Welsh et al., 1991).

However, little is known about the sources of individual differences in executive functions. Within a biological framework, a common hypothesis is that the development of executive functions is the result of the maturational unfolding of the genetic program (e.g., Diamond, 2002; Garon et al., 2008) and that individual differences in executive functions are largely genetically determined (Friedman et al., 2008). By contrast, according to a social relational approach (Carpendale and Lewis, 2006; Lewis and Carpendale, 2009), the development of executive functions involves social and biological factors, and social interaction plays an important role in explaining individual differences in executive functions (e.g., Carlson, 2009). Several studies exist that explore whether and how social factors influence individual differences in executive functions. Among these, parenting scaffolding has been examined. According to Wood et al. (1976) and Bernier et al. (2010) scaffolding can be understood in terms of parental support of a child’s autonomous problem solving, whether verbally or physically mediated. Indeed, a handful longitudinal studies showed substantial relations between parental scaffolding in early childhood and later executive functions (Landry et al., 2002; Hughes and Ensor, 2009; Bernier et al., 2010; Hammond et al., 2012).

In sum, higher levels of executive functions have been associated with academic achievement. Furthermore, pioneer work suggests a social component being responsible for the development of individual differences in executive functions.

### MOTIVATION STYLES IN CHILDREN AND ACADEMIC ACHIEVEMENT

Despite the widely acknowledged contribution of cognitive abilities to academic achievement, research data indicate consistently that cognitive abilities alone fail to have enough power predicting educational success. Many factors seem to influence how children and adolescents in school succeed. Among these, motivation is considered to play a crucial role. One widely accepted and well investigated theoretical and empirical approach to motivation is self-determination theory (SDT; Deci and Ryan, 1985; Ryan and Deci, 2000). SDT proposes different types of motivation reflecting different levels of autonomous self-regulation. On a continuum from the lowest to the highest levels of self-regulation, there are external regulation, introjected regulation, identified regulation, and intrinsic motivation. Intrinsic motivation refers to the incentive to perform an activity for its own sake, for the inherent interest in that activity (Ryan and Deci, 2000), while extrinsic motivation refers to the incentive to perform an activity for instrumental reasons that are separate from the activity (Deci et al., 1991). High levels of autonomous self-regulated motivation such as intrinsic motivation are fostered by the experience of three fundamental basic psychological needs, that is, the needs for autonomy (i.e., experiencing a sense of volitional and psychological freedom), the need for competence (i.e., experiencing personal effectiveness), and the need for social relatedness (i.e., experiencing closeness and mutuality in interpersonal relationships). In contrast, little experience of these needs will results in decreased motivation and decreased overall well-being with the result of little self-determined behavior. This view is based on strong empirical support indicating how self-determined behavior is associated with diverse positive consequences in various life domains (Vallerand, 1997; Deci and Ryan, 2000). The strongest support for SDT stems from investigations in the educational domain showing several positive outcomes of self-regulated motivation styles to learning, such as higher feelings of self-perceived and teacher-perceived academic competence (Fortier et al., 1995), the use of optimal learning strategies (Yamauchi et al., 1999), the use of less defensive coping styles (Ryan and Connell, 1989), greater motivation to attend and participate in class or higher school grades (Reeve et al., 1999; Black and Deci, 2000; Vansteenkiste et al., 2004; Guay et al., 2008). In sum, highly autonomous self-regulatory motivation styles depend strongly on the experience of autonomy, competence, and social relatedness.

### TEACHERS’ SELF-REGULATORY SUPPORT AND ACADEMIC ACHIEVEMENT

However, in contrast to stable personality traits, expressions of motivation show great inter-individual differences. Social or environmental factors such as teacher behavior can influence how students motivationally respond and act in school. According to the SDT model of the teacher–student relationship, motivation styles strongly depend on high autonomy supportive versus high controlling environments. Autonomy support refers to teachers’ promotion of volitional functioning and teachers’ fostering of a sense of initiative and interest in students. Autonomy support is contrasted with a controlling interpersonal style, that is, a style where teachers ignore the students’ perspective and pressure the students to think, act, or feel in a particular way (Deci and Ryan, 1994; Reeve, 2009; Grolnick, 2013). Respectively, students who perceive their teachers as autonomy supportive are more likely to experience higher levels of intrinsic motivation due to a satisfaction of basic needs. Empirical research has consistently shown that an autonomy-supportive teaching style is positively associated with more school engagement (Assor et al., 2002), higher grades and better school adjustment (Ryan et al., 1994; Fortier et al., 1995; Guay and Vallerand, 1997; Wentzel, 2002; Patrick et al., 2012), higher classroom engagement (Reeve et al., 2002; Tsai et al., 2008), more positive emotionality (Reeve and Jang, 2006), creativity (Amabile, 1985), and persistence in school (Vallerand et al., 1997).

In contrast, teachers’ support is negatively associated with students experience stress (Torsheim and Wold, 2001; Assor et al., 2005). But moreover: research has provided evidence for a positive relationship between teachers’ autonomy support and higher levels of self-determined learning (Grolnick et al., 1991; Reeve et al., 1999). In contrast, controlling environments, such as engagement-contingent reward (Deci, 1971) or deadline and surveillance (Deci et al., 1999) are associated with a feeling of coercion that diminishes motivation. In sum, environments that support autonomous self-regulated learning such as autonomous supportive teachers influence students’ motivation orientation and degree of self-regulated learning strategies.

In order to add to the existing literature, we investigated the interplay between executive functions in school children and psychological and social factors such as motivation styles and teachers’ autonomy support, respectively. From the standpoint of research on learning and developmental psychology, there is a growing need to increase our understanding what affects inter-individual differences in the development of executive functions as a core requirement for motivation. To our knowledge, to date only one study examined the interplay of executive functions and motivation in school children. Mizuno et al. (2011) found that decrease in capacity for verbal working memory was associated with the prevalence of decrease in intrinsic academic motivation among junior high school students. According to Mizuno et al. (2011) intrinsic academic motivation must engage the working memory system to relate achievements to an ultimate goal. Especially while learning at school, the working memory maintains a limited amount of currently relevant information so that it is available for immediate use. Thus, function of working memory (which is included in executive function) permits goal-directed behavior.

In the present study, we implemented a cross-sectional study design within a sample of primary and junior high school children and administered different executive function tests and self-report scales measuring motivation styles and autonomous self-regulation support by teachers. We expect motivation styles in children and teachers’ autonomy support to act as modulators of inter-individual differences in the development of executive functions, i.e., individual expressions of intrinsic motivation styles would be associated with higher levels of executive functions, and vice versa, while individual expressions of highly controlling behavior would be associated with lower levels of executive functions in children and adolescents, and vice versa.

## MATERIALS AND METHODS

### PARTICIPANTS

In a cross-sectional study we tested and questioned 208 children and consulted 150 teachers. We only consulted those teachers that were regularly in contact with the tested children either as class teacher or as specialist subject teacher. 208 children of different ages and from different schools participated: we investigated 50 primary school children (grades: 3 and 4, mean age: 9.18, SD: 0.774, range: 8–11 years, 23 boys/27 girls), 83 junior high school children attending so called middle schools (grades: 5 and 6, mean age: 11.29, SD: 0.877, range: 10–14 years, 44 boys/39 girls), and 75 junior high school children attending so called Gymnasien (grades: 5 and 6, mean age: 11.07, SD: 0.800, range: 10–13 years, 36 boys/39 girls). In Saxony, two types of junior high schools exist: middle schools and Gymnasien. After running through primary school most of all children were allocated to Gymnasien (63%; see PISA-Konsortium Deutschland, 2008). One third, the cognitively less powerful children, transfer to middle schools. All children volunteered for participation after an informative meeting. Children and parental informed consent to participate were obtained in writing prior to data collection. In addition, 58 primary school teachers (mean age: 47.69, SD: 9.349, range: 23–63 years, 50 female/8 male), 49 junior high school teachers of middle schools (mean age: 47.88, SD: 7.336, range: 27–61 years, 42 female/7 male), and 43 junior high school teachers of Gymnasien (mean age: 46.05, SD: 9.127, range: 26–58 years, 35 female/8 male) were surveyed. The teachers volunteered for participation after an informative meeting and signed an informed consent. The study was approved by the local internal review board of the Medical Faculty of the University of Ulm.

### PROCEDURE

Children attended regular school and were tested at times were regular school lessons were usually held, so they did not have to appear at additional time. In order to carry out tests on executive function children had to move to a computer room and were tested in small groups. Children and teachers questionnaires were distributed at school. Children got a short instruction by a member of the project team and completed the form in the classroom. Teachers had the opportunity to answer their questionnaire at home and handed it back after a few days.

### MATERIAL

#### Executive function tests

**FIGURE 1:**
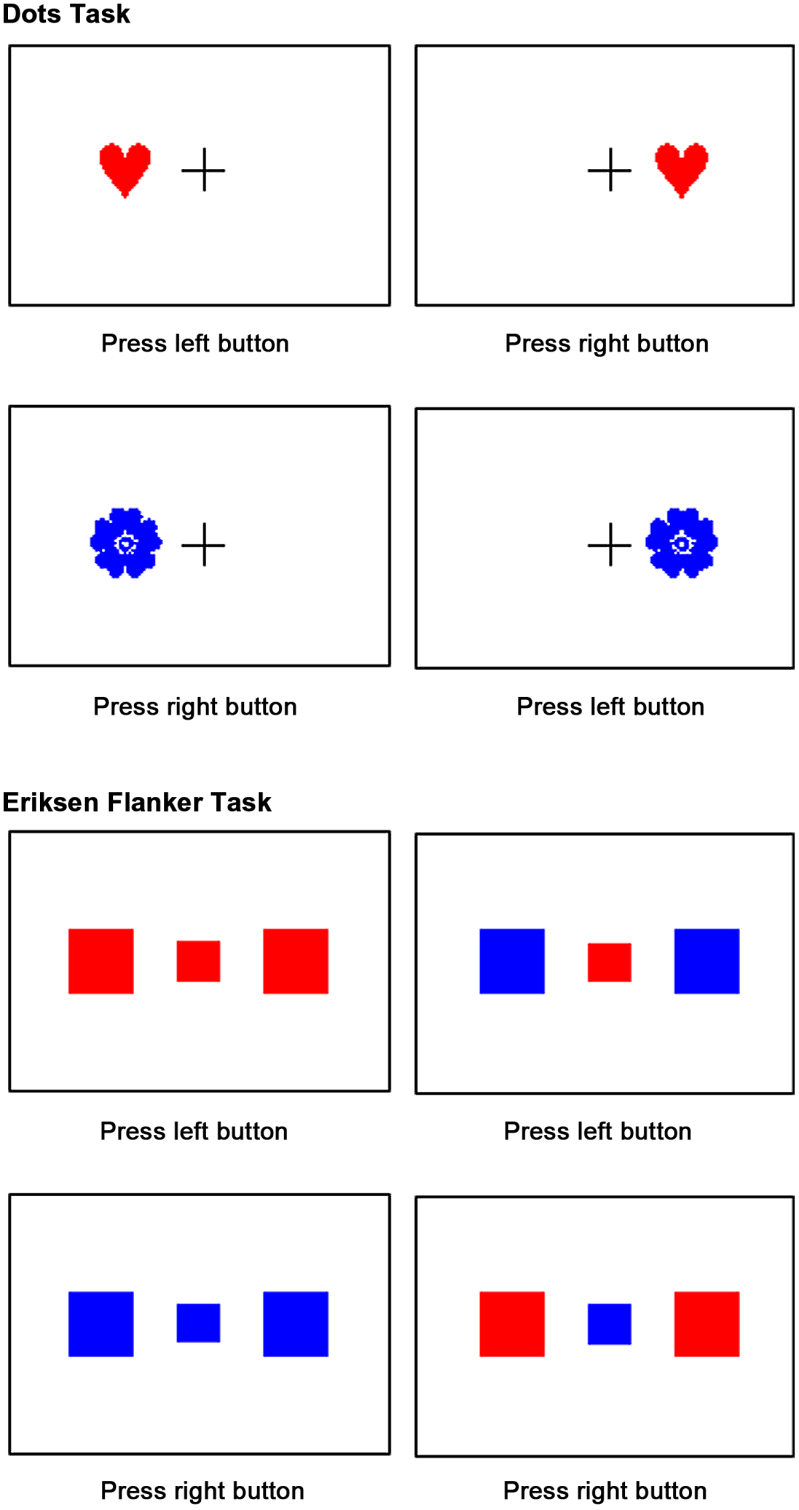
**Examples of the different trials of the two executive function tests**.

To assess executive functions in children we implemented the dots task (Davidson et al., 2006) and the Eriksen flanker task (Eriksen and Eriksen, 1974) as computer based tests (see **Figure 1**). Both tasks are appropriate for ages 4 through adults.

In all conditions of the dots task a red heart or blue flower appeared on the right or left of a fixation cross. Children run through three conditions: in the congruent condition solely red hearts were presented and the children were told to press the mouse button on the same side as the stimulus. In the incongruent condition solely blue flowers were presented. Now the children were told to press the mouse button on the opposite side as the stimulus. This condition requires that children inhibit the tendency to respond on the same side (the so-called Simon effect, Lu and Proctor, 1995). In the third condition, the mixed condition, congruent and incongruent trials were intermixed in equal shares. The mixed condition was the most challenging, since it required that children remembered two rules (“press on the same side as the red heart” and “press on the side opposite the blue flower”) and inhibit the tendency to respond on the same side as the stimulus on one-half of the trials. First, children handled at least 20 congruent, 20 incongruent, and 20 mixed practice trials. In order to control for floor-effect, the test started as soon as a child passed the mixed practice trials with less than five errors. During the test children performed a block of 40 trials in the congruent condition, a block of 40 incongruent trials and 40 mixed trials. The inter stimulus interval was 500 ms, stimuli were presented for 750 ms. Feedback was provided during the practice but never during performance of the test.

During the Eriksen flanker task children focused on the color of a small red or blue rectangle (i.e., the target) presented in the center of the screen. The target stimulus was flanked by two rectangles that appeared 4.5 cm to the left and to the right of the target that were either red or blue. Height and width of each flanker was three times that of the target. Target and flanker were displayed simultaneously. In the congruent condition both, target and flanker, matched in color. In the incongruent condition the flanker were blue when the target was red and vice versa. Children were instructed to respond depending on the color of the target by pressing the left or right mouse button with their dominant hand. Stimuli were presented until button press, the inter stimulus interval was 500 ms. Appearance of the four combinations of target and flanker color was equiprobable (blue–blue, blue–red, red–blue, red–red). Prior to the test, children completed 60 practice trials (half congruent, half incongruent) and the final test comprised 40 congruent and 40 incongruent trials, with randomized order of trials.

All executive function tests were conducted via computer presentation positioned at eye level and at a distance of 50 cm from the children. The mouse was placed on a table in front of the children at a comfortable distance. The response keys were the left and right mouse key marked with different stickers. In general, children were instructed to respond as quickly and accurately as possible.

Both tasks assess all three major executive function domains – inhibition, working memory, and cognitive flexibility (see Diamond et al., 2007a,b; Diamond, 2013). The Dots task requires that subjects remember two rules in working memory (for hearts press on the same side as the stimulus; for flowers press on the side opposite the stimulus). In the congruent condition, one rule applies (“press the button on the same side as the heart”). The incongruent condition also requires remembering a rule (“press the button on the side opposite the flower”) but in addition it requires inhibiting the tendency to respond on the side where a stimulus appears. In the mixed condition two abstract rules have to be held in mind (=working memory), cognitive flexibility is needed to switch between rules, and inhibition is needed on incongruent and switch trials. Both the congruent and incongruent Flanker conditions require inhibition and some memory of the rules (though memory aids are provided). The incongruent condition also requires inhibition of what they have just practiced and thus the cognitive flexibility to change the focus of attention and stimulus-response mappings.

The output of the executive function tests was prepared as follows: response times faster than 200 ms were considered too fast to be in response to the stimulus and excluded from further analyses of error rate (accuracy) and reaction time (speed). The error rate was calculated by dividing the number of incorrect responses by the sum of correct plus incorrect responses. The reaction time was calculated for correct responses only. The dependent measures mean reaction times and mean error rate were computed separately for each participant and each condition.

#### Children questionnaire

The academic self-regulation questionnaire (SRQ-A) by Connell and Ryan (1986) concerns the reasons why children do their school work. The questionnaire asks why the respondent does a behavior (or class of behaviors) and then provides several possible reasons that have been preselected to represent four different styles of motivation, namely intrinsic motivation, identified regulation, introjected regulation, and external regulation. Intrinsic motivation is the most self-determined motivation style. Intrinsic motivated children pursue an activity out of interest and enjoyment and without external contingencies. Identified regulated children undertake an activity because they accept the value of the activity. Introjected and external regulation styles are the less self-determined forms: introjected regulated children partake in an activity because of internal pressures such as guilt or shame. External regulated children pursue an activity because of external pressures or incentives. Respondents rate the degree of appropriateness of each of the provided reasons on a four-point scale. We computed mean scale values for each participant and each subscale were computed. To keep the complexity of our design within reasonable limits, we focus on the extreme scales intrinsic motivation and external regulation.

The scale was developed for students in late primary and secondary school and translated in German using the systematic back-translation technique. Analyses of psychometric properties of the original version revealed moderate to high levels of internal consistency for the four subscales ranging from 0.62 to 0.82 (Connell and Ryan, 1986). A series of pre-tests evaluating our German translation were conducted with a total of 59 children from primary and secondary school, in order to assess the comprehension of the translated and adapted questionnaire. The data obtained by the pre-tests revealed good psychometric properties such as: Cronbach’s alphas (intrinsic motivation: 0.88; identified regulation: 0.83; introjected regulation: 0.83; external regulation: 0.79), item-difficulty [= the average score on an item divided by the highest number of points for any one alternative; ideal item-difficulty levels are ranging between 0.20 and 0.80 (see Bühner, 2011); the item-difficulty levels of the German version of the SRQ-A range between 0.36 and 0.83] and item-discrimination [= Pearson product moment correlation between student responses to a particular item and total scores on all other items of the scale; ideal discrimination indices are above 0.30 (see Bühner, 2011); the discrimination indices of the German version of the SRQ-A range between 0.35 and 0.74]. The psychometric properties obtained by the current sample are as follows: Cronbach’s alphas (intrinsic motivation: 0.85; identified regulation: 0.86; introjected regulation: 0.79; external regulation: 0.77), item-difficulty (ranging between 0.44 and 0.86) and item-discrimination (ranging between 0.33 and 0.74).

#### Teacher questionnaire

The problems in schools questionnaire by Deci et al. (1981) - German translation by Martinek (2007) - assesses whether teachers tend to be controlling versus autonomy supportive while interacting with their students. The measures are composed of eight vignettes, each of which is followed by four ways of responding to the situation – one is highly controlling, one is moderately controlling, one is moderately autonomy supportive, and one is highly autonomy supportive. Respondents rate the degree of appropriateness of each of the four options (on a seven-point scale) for each of the eight situations. According to the manual, we computed mean scale values for each participant and each subscale. Again we concentrate our analyses on the extreme scales, highly autonomy supportive and highly controlling. Alpha reliabilities for the subscales of the English/German version of the problems in school questionnaire were: highly controlling = 0.73/0.75 and highly autonomy supportive = 0.80/0.65 (for the Englisch original version see Deci et al., 1981, for the German version see Martinek, 2007).

### STATISTICAL ANALYSES

Question 1: Do the executive function tests work?

The executive function tests (dots task and Eriksen flanker task) were analyzed by within-subjects ANOVAs in order to verify, that the student’s performances were systematically influenced by the different task conditions. As dependent variables we considered both reaction times and error rate. The independent variable is the task condition (Eriksen flanker task: congruent and incongruent, dots task: congruent, incongruent and mixed). For the dots task the independent variable task condition covers three levels, therefore a significant main effect task condition is further examined by pairwise contrasts.

Question 2: How does teachers’ autonomy support relate to children’s motivation?

Specific influences on the dependent variables intrinsic motivation and external regulation were analyzed by linear mixed models. Considering that the present study was conducted in a school setting, where students are nested in schools, we used a multilevel modeling approach - the SPSS mixed procedure (SPSS, 2005). The school level was included as random effect to account for common variance. In addition to the random effect “schools,” the teacher level had been introduced as random effect, too. But when estimating the mixed model convergence failed. Presumably the variance of this random effect has been too small. Therefore, the present and all following analyses were computed without the random effect teacher level. Random-intercept models were estimated in which the intercepts were allowed to vary randomly but with fixed effects for all predictor variables. The method of estimation applied for all models was restricted maximum likelihood. The two subscales of the problems in schools questionnaire, highly autonomy supportive and highly controlling, were added as fixed effects. To account for possible bias, gender and type of school were included as fixed effects, too.

Question 3: How does children’s motivation relate to their executive functions?

Specific influences on the executive functions were analyzed by linear mixed models. Again a multilevel modeling approach, linear mixed models, was used. The school level was included as random effect to account for common variance. The method of estimation was restricted maximum likelihood. The dependent variable executive function was tested by introducing the difference scores of reaction times and error rates following incongruent trials minus congruent ones. These difference scores display the additional allocation of executive function capacities while coping with challenging tasks. The analyses were computed separately for the difference scores of error rate and reaction time and separately for the dots and the Eriksen flanker task. The two subscales of the SRQ, intrinsic motivation and external regulation, were added as fixed effects. To account for possible bias, gender and type of school were included as fixed effects, too.

Question 4: How does teachers’ autonomy support relate to their children’s executive functions?

In our fourth analyses linear mixed models were used to disclose systematic effects of the teachers’ autonomy support on their children’s executive functions. Again, school level was included as random effect. The dependent variable executive function was tested by introducing the difference scores of reaction times and error rates following incongruent trials minus congruent ones. Analog to the third analysis computations were done separately for the difference scores of error rate and reaction time and separately for the dots and the Eriksen flanker task. The problems in school questionnaire highly autonomy supportive and highly controlling were added as fixed effects. To account for possible bias, gender and type of school were included as fixed effects, too.

## RESULTS

**Table 1** shows descriptive statistics for the subscales of the SRQ, the subscales of the problems in school questionnaire and the two executive function tests, the dots and the Eriksen flanker task.

**Table 1:** Descriptive statistics.

	Mean	SD	Minimum	Maximum
**Dots task**
Reaction time: congruent	440.00	57.64	309.96	586.23
Reaction time: incongruent	473.54	67.66	284.20	642.99
Reaction time: mixed	649.67	101.04	369.42	902.41
Error rate: congruent	0.03	0.08	0.00	0.67
Error rate: incongruent	0.07	0.19	0.00	1.00
Error rate: mixed	0.12	0.13	0.00	0.62
Reaction time: incongruent – congruent	33.08	49.38	-154.10	167.65
Reaction time: mixed – congruent	209.67	82.25	-77.16	419.65
Error rate: incongruent –congruent Error rate: mixed – congruent	0.04 0.09	0.19 0.12	-0.59 -0.32	1.00 0.62
**Eriksen flanker task**		
Reaction time: congruent	585.98	90.20	364.33	874.00
Reaction time: incongruent	619.76	135.18	331.50	1526.00
Error rate: congruent	0.09	0.17	0.00	0.94
Error rate: incongruent Reaction time: incongruent – congruent Error rate: incongruent – congruent	0.11 33.78 0.01	0.18 91.83 0.06	0.00 -192.91 -0.21	0.98 862.67 0.20
**Self-regulation questionnaire**
Intrinsic motivation	1.68	0.71	0.00	3.00
External regulation	2.05	0.55	0.56	3.00
**Problems in school questionnaire**
Highly controlling	1.73	0.57	0.38	2.88
Highly autonomy supportive	4.73	0.72	3.25	5.88

With regard to reaction time and error rate, performance in the dots task was best in the congruent condition, intermediate in the incongruent one, and worst in the mixed condition. Performance in the incongruent and mixed condition can also be viewed as a deviation from performance in the congruent condition (incongruent minus congruent, mixed minus congruent), thus taking into account baseline performance. This deviation was greater for performance in the mixed condition than in the incongruent one. The mean scores in the Eriksen flanker task reveal very similar: reaction time is faster and error rate is lower in the congruent condition than in the incongruent. The descriptive statistics for the SRQ disclose that children assess themselves to be more often external regulated then to be intrinsic motivated. Intrinsic motivation and external regulation did not correlate (Pearsons *r* = 0.021; *p* = 0.760). The participating teachers rate themselves acting much more autonomy supportive than controlling. Highly autonomy supportive and controlling behavior did not correlate (Pearsons *r* = -0,115, *p* = 0.162).

### DO THE EXECUTIVE FUNCTION TESTS WORK?

Analysing the reaction time of the dots task, the within-subject ANOVA reveals a significant main effect task condition [*F*(2,334) = 922.171; *p* < 0.001; $\eta_{p}^{2}$ = 0.847]. Contrasts comparing the congruent and the incongruent [*F*(1,167) = 75.367; *p* < 0.001; $\eta_{p}^{2}$ = 0,311], as well as the congruent and the mixed condition [*F*(1,167) = 1114.771; *p* < 0.001; $\eta_{p}^{2}$ = 0.870], show that the congruent trials were followed by the fastest reaction times and the mixed trials were followed by the slowest reaction times with the incongruent trials lying in between. Analysing the error rate of the dots task, the within-subject ANOVA discloses again a significant main effect task condition [*F*(2,338) = 29.687; *p* < 0.001; $\eta_{p}^{2}$ = 0,149]. Contrasts reveal the following pattern: the error rate following congruent trials is significant lower compared to incongruent [*F*(1,169) = 6.642; *p* = 0.011; $\eta_{p}^{2}$ = 0.038] and mixed [*F*(1,169) = 97.250; *p* < 0.001; $\eta_{p}^{2}$ = 0.365] trials. The within-subject ANOVAs of the Eriksen flanker task discloses a significant difference between the incongruent and congruent trials with regard to reaction times [main effect task condition: *F*(1,169) = 23.002; *p* < 0.001; $\eta_{p}^{2}$ = 0.120; with faster reaction times following congruent trials] as well as with regard to error rate [main effect task condition: *F*(1,169) = 7.500; *p* = 0.007; $\eta_{p}^{2}$ = 0.042; with lower error rates following congruent trials].

These results confirm that the student’s responses were systematically stressed by the task conditions. Further analyses consider the difference scores of reaction time and error rate following incongruent trials minus congruent ones. These difference scores take baseline performance into account and display the additional allocation of executive functions while coping with tasks of differing demands (see Davidson et al., 2006).

### HOW DOES TEACHERS’ AUTONOMY SUPPORT RELATE TO CHILDREN’S MOTIVATION?

The effects of teacher’s behavior on student’s motivation styles were examined using linear mixed models.

The results can be seen in **Table 2**, separately for intrinsic motivation (left part) and external regulation (right part). Children’s motivation style is influenced by their teachers’ autonomy support. Children, whose teachers rate themselves being highly autonomy supportive, show higher intrinsic motivation (*B* = 0.17), whereas children, whose teachers assess themselves being highly controlling, disclose higher external regulation styles (*B* = 0.20). Motivation is influenced by gender and school type. Girls reveal higher intrinsic motivation scores compared to boys (*B* = 0.24) and students of middle schools show lower intrinsic motivation scores (*B* = -0.57) and higher external regulation scores (*B* = 0.33) compared to primary school children.

**Table 2:** Results [*B* = estimates of fixed effects; SE(*B*) = standard errors] of linear mixed model analyses predicting children’s motivation by teachers’ autonomy support.

Predictors	Self-regulation questionnaire			
	Intrinsic motivation		External regulation	
	*B*	SE(*B*)	*B*	SE(*B*)
**Child**		
Gender (ref.: boys)	0.24**	0.10	-0.06	0.08
**School type (ref.: primary schools)**		
Middle schools	-0.57**	0.21	0.33**	0.15
Gymnasien	-0.26	0.25	-0.11	0.17
**Problems in school questionnaire**		
Teacher: highly autonomy supportive	0.17**	0.07	-0.07	0.06
Teacher: highly controlling	0.01	0.11	0.20**	0.08
*R*^2^	0.73		0.88	
ICC	0.20		0.16	

**p < 0.05; *R*^2^, reduction in variance estimates for the within schools portions of the model; ICC, proportion of variance between schools compared to the total variation; all continuous predictors were grand-mean centered before being included in the model.

**Table 2** also provides information about the reduction in variance estimate (*R*^2^) for the within-school portions of the model. All fixed effects together account for about 73% of the within-school variability in student’s intrinsic motivation and for 88% of the within-school variability in student’s external regulation. Both values of *R*^2^ are quite high, showing that the data fits well to the regression model. The proportions of variance between schools compared to the total variation (=ICC) add up to 20% predicting intrinsic motivation and 16% predicting external regulation. Heck et al. (2010) suggest that the development of a multilevel model is warranted if the ICC is higher than 5%.

### HOW DOES CHILDREN’S MOTIVATION RELATE TO THEIR EXECUTIVE FUNCTIONS?

**Table 3** displays the results of the linear mixed model analysis of how children’s motivation is related to their executive functions. On the left side of **Table 3**, the results predicting error rate and reaction time difference scores of the dots task are depicted and on the right side, the results predicting error rate and reaction time difference scores of the Eriksen flanker task are depicted. As can be seen, high intrinsic motivation scores came along with low error rate difference scores (dots task: *B* = -0.021) and high external regulation scores are accompanied by high error rate difference scores (dots task: *B* = 0.036; Eriksen flanker task: *B* = 0.040). Thus, intrinsically motivated children reveal better executive functions compared to external regulated children. Reaction times are not affected by the motivational style. The analysis of the dots task also shows, that girls reveal slower reaction times compared to boys (*B* = 24.23) and junior high school students [middle school students (*B* = -52.74) as well as students from Gymnasien (*B* = -53.99)] show faster reaction times compared to primary school children.

**Table 3:** Results [*B* = estimates of fixed effects; SE(*B*) = standard errors] of linear mixed model analyses predicting the executive function difference scores incongruent minus congruent by children’s motivation.

Predictors	Dots task: incongruent minus congruent				Eriksen flanker task: incongruent minus congruent			
	Error rate		Reaction time		Error rate		Reaction time	
	*B*	SE(*B*)	*B*	SE(*B*)	*B*	SE(B)	*B*	SE(*B*)
**Child**
Gender (ref.: boys)	-0.023	0.018	24.23*	12.33	-0.001	0.015	12.37	14.44
**School type (ref.: primary schools)**
Middle schools	-0.019	0.028	-52.74**	19.40	-0.002	0.029	-4.09	22.71
Gymnasien	-0.038	0.031	-53.99**	20.97	-0.041	0.026	-19.37	24.55
**Self-regulation questionnaire**
Intrinsic motivation	-0.021*	0.013	-5.21	8.89	-0.001	0.011	-8.87	10.40
External regulation	0.036**	0.017	16.72	11.64	0.040**	0.014	12.30	13.62
*R*^2^	0.51		0.23		0.60		0.19	
ICC	0.09		0.15		0.06		0.23	

**p < 0.05; *p < 0.10; *R*^2^, reduction in variance estimates for the within schools portions of the model; ICC, proportion of variance between schools compared to the total variation; all continuous predictors were grand-mean centered before being included in the model.

Values of *R*^2^ range from 19 to 60%. The linear mixed models predicting error rate difference scores show a better model fit (51 and 60%) compared those predicting reaction time difference scores (23 and 19%).

### HOW DOES TEACHERS’ AUTONOMY SUPPORT RELATE TO THEIR CHILDREN’S EXECUTIVE FUNCTIONS?

The results depicting the linear mixed model analysis of the effects of the teacher behavior on their children’s executive functions can be seen in **Table 4**. The left side of **Table 4** presents the results concerning error rate and reaction time of the dots task and the right side presents the results concerning error rate and reaction time of the Eriksen flanker task. Children, whose teachers rate themselves being highly autonomy supportive, show better executive functions [defined as lower error rate difference scores performing the dots (*B* = -0.037) and the Eriksen flanker task (*B* = -0.014)]. Between teachers’ highly controlling and their children’s motivation style no impact reveals significant. Reaction times are not affected by teachers’ autonomy support. Girls reveal lower error rates compared to boys (dots task: *B* = -0.098; Eriksen flanker task: *B* = -0.014). For the dots task junior high school students [middle school students (*B* = -39.03) as well as students from Gymnasien (*B* = -52.00)] show faster reaction times compared to primary school children.

**Table 4:** Results [*B* = estimates of fixed effects; SE(*B*) = standard errors] of linear mixed model analyses predicting the executive function difference scores incongruent minus congruent by teachers’ autonomy support.

Predictors	Dots task: incongruent minus congruent				Eriksen flanker task: incongruent minus congruent			
	Error rate		Reaction time		Error rate		Reaction time	
	*B*	SE(*B*)	*B*	SE(*B*)	*B*	SE(*B*)	*B*	SE(*B*)
**Child**
Gender (ref.: boys)	-0.098**	0.030	12.18	7.48	-0.014*	0.008	13.97	14.80
**School type (ref.: primary schools)**
Middle schools	-0.032	0.059	-39.03**	14.21	-0.009	0.016	-4.97	25.89
Gymnasien	-0.062	0.054	-52.00**	13.03	-0.013	0.015	-27.27	28.27
**Problems in school questionnaire**
Teacher: highly autonomy supportive	-0.037*	0.022	-5.85	5.33	-0.014**	0.006	-3.42	10.55
Teacher: highly controlling	0.018	0.027	4.19	6.82	0.002	0.008	3.62	13.37
*R*^2^	0.63		0.22		0.59		0.12	
ICC	0.05		0.06		0.08		0.08	

**p < 0.05; *p < 0.10; *R*^2^, reduction in variance estimates for the within schools portions of the model; ICC, proportion of variance between schools compared to the total variation; all continuous predictors were grand-mean centered before being included in the model.

Values of *R*^2^ range from 12 to 63%. Again, the linear mixed models predicting error rate difference scores show a better model fit (63 and 59%) compared those predicting reaction time difference scores (22 and 12%).

## DISCUSSION

The present study pursues the following objectives: first, the interplay of student’s motivation style and their teacher’s autonomy support is investigated. Second, systematic impacts of the aforementioned factors on children’s executive functions are addressed.

The analysis reveals that teacher’s autonomy supportive or controlling behavior is associated with their children’s motivation style. High autonomy supportive behavior is positively related to intrinsic motivation whereas high controlling behavior is positively related to external regulation. This result is in accordance with many others studies (Deci et al., 1991; Reeve et al., 2004; Soenens and Vansteenkiste, 2005; Reeve, 2006). Stroet et al. (2013) systematically reviewed 71 empirical studies on the effects of autonomy supportive teaching on student’s motivation and engagement for school and found a clear positive association. But, as Stroet et al. (2013) note, most of these studies used student perceptions to measure autonomy supportive teaching. Studies using teacher perceptions are missing. Here the present study can make an important contribution, since we found positive associations of teacher’s self-evaluation of their autonomy supportive or controlling behavior and their student’s motivation styles.

The present data further discloses that student’s motivation styles are related to their executive functions. Students who showed lower error rate difference scores while performing the executive function tasks scored higher in intrinsic motivation and students with higher error rate difference scores more often made use of external regulation strategies. However, the causal relationship between these variables is still unclear. It is possible that external regulation strategies prevent development and training of executive functions. It is likewise possible that student’s low executive functions capacities require external regulation strategies. Future research should tackle this.

Doing well in executive function tasks requires that children react to errors when they occur. To minimize future errors they have to learn from them. Past research has shown that autonomy improves self-regulation because it fosters openness to failures (Koestner and Zuckerman, 1994; Hodgins and Liebeskind, 2003; Weinstein et al., 2011). Autonomously acting people “…are less defensive and ego-protective and tend to openly acknowledge negative affect or criticism and personal shortcomings” (cited from Legault and Inzlicht, 2013, p. 125). Keeping in mind that autonomy and motivation are interconnected, we suppose that intrinsic motivation increases attention in performance monitoring during the executive function tasks and improves the reaction to one’s errors. External regulation, on the other hand, is associated with decreased attention and error receptivity, resulting in higher error rates. In line with our result, neuroscientists (Fisher et al., 2009; Legault and Inzlicht, 2013) report significant positive correlations of intrinsic motivation with an event related potential, the error-related negativity. This error-related negativity reflects an error detection system that monitors performance and detects incongruity between intended and actual responses (Holroyd and Coles, 2002).

The last analysis focused the impact of teachers’ autonomy support on their children’s executive functions. Teachers that support autonomy are related to students that produce lower error rate difference scores while performing executive functions tests. Given this significant relationships, from an applied perspective, it is important for teachers to prescind from controlling behavior and to be advised to teach in a more autonomy-supportive fashion. So what can teachers do to enhance their student’s motivation and executive functions? Several studies (Reeve et al., 1999; Black and Deci, 2000; Assor et al., 2005) revealed that autonomy supportive environments promote the salience of intrinsic goals (e.g., personal growth) and minimize external incentives (e.g., money) and threats. In order to grasp the importance of an intrinsic goal, students have to freely choose tasks that they perceive as consistent with their goals and interests. Teacher may support this by using phrases such as, “you can,” “you might,” “if you choose,” and “we ask you to,” instead of phrases such as “you should,” “you have to,” “you’d better,” and “you must.” They further may support autonomy by creating opportunities for students to work in their own way. Teachers should not keep possession of and monopolize the learning material they rather should arrange learning materials so students can “serve themselves.” Thus, instead of passively watching and listening students have to organize their own learning process in a self-directed active way. The teacher’s task is to offer progress-enabling hints when students seem stuck, to respond to student’s questions and comments and to encourage effort and persistence.

In addition to our major results the present analyses further revealed that children’s gender and school type are associated with their intrinsic motivation score. It appears that girls display more intrinsic motivation than boys. Usually school-related intrinsic motivation is investigated in a domain-specific way. Most of these studies found that girls achieve higher intrinsic motivation scores for languages and boys for mathematics (e.g., Jacobs et al., 2002; Spinath et al., 2006; Freudenthaler et al., 2008). The present study has questioned intrinsic motivation of boys and girls in a general school-related way. Thus, high scores indicate that girls show stronger willingness to learn and to do well in school (i.e., to achieve good grades and to behave in a more agreeable way). Our result might be a potential mediator of the so-called gender gap in educational achievement (Salisbury et al., 1999), but needs to be assessed in further research. The present study showed that school type is related to intrinsic motivation, whereby older children, who are visiting middle schools and Gymnasien, show a general decrease of intrinsic motivation compared to younger ones, who are visiting primary schools. This result is in line with others (Harter, 1981; Gottfried et al., 2001; Lepper et al., 2005). The decrease in intrinsic motivation is explained by an increasingly controlling school environment.

### LIMITATIONS

One limitation concerns the study design. We did not have the opportunity to observe the schools by a longitudinal study, all data was raised at a single measurement point. Thus, we are not in a position to make assumptions about potential causal links between the investigated variables. A second limitation is that we did not control for IQ. On average more children with higher IQ are allocated for Gymnasien than middle schools. Since we did not control for IQ we cannot exclude a modulating effect of such a general intelligence variable.

## Conflict of Interest Statement

The authors declare that the research was conducted in the absence of any commercial or financial relationships that could be construed as a potential conflict of interest.
